# Effects of canagliflozin on cardiovascular disease risk factors in patients with type 2 diabetes: a systematic review and meta-analysis

**DOI:** 10.1186/s12902-025-01984-3

**Published:** 2025-07-02

**Authors:** Hossein Aftabi, Reyhaneh Aftabi

**Affiliations:** 1 Department of Biology, Faculty of Sciences, Shahid Bahonar University of Kerman, Kerman, Iran; 2 Social Determinants on Oral Health Research Center, Kerman University of Medical Sciences, Kerman, Iran

**Keywords:** Canagliflozin, Type 2 diabetes mellitus, Cardiovascular disease, Hemoglobin level, Body mass index, Systolic blood pressure, Placebo

## Abstract

**Background:**

Canagliflozin or sodium-glucose co-transporter 2 inhibitor (SGLT2i) is considered as an authorized therapeutic drug for treatment of patients with type 2 diabetes mellitus (T2DM). This study reviews and evaluates the effects of Canagliflozin on Hemoglobin (HbA1c), Body Mass Index (BMI) and Systolic Blood Pressure (SBP).

**Methods:**

This fixed-effects systematic review and meta-analysis are based on 38 comprehensive literature survey and statistical analysis of selected references that explore the effect of canagliflozin in patients having cardiovascular disease (CVD) and T2DM. The data were analyzed and interpreted at 95% Confidence Interval with reference to placebo-controlled randomized controlled trails (RCTs).

**Results:**

The effects of canagliflozin at 100 and 300 doses slightly reduced Hemoglobin A1c (HbA1c) and Body Mass Index (BMI) without significant differences with placebo [HbA1c at 100 mg, effect size: -0.005, Confidence Interval of 95% = -0.04 to 0.03, (*P* = 0.79), at 300 mg, effect size: -0.03 (-0.11 to 0.05), (*P* = 0.43), BMI at 100 mg, effect size: -0.01 (-0.04 to 0.02), (*P* = 0.57) and at 300 mg, effect size was 0.02 (-0.05 to 0.10), (*P* = 0.55)]. At 100 mg dose, canagliflozin lowers systolic blood pressure compared to that of placebo (effect size: -0.03 (-0.07, 0.00), (*P* = 0.06)]. These data up to date reveal that the most significant effective role of canagliflozin in patients having T2DM is to reduce the systolic blood pressure.

**Conclusion:**

This systematic review and meta-analysis highlight that although canagliflozin does not project significant decrease on BMI and HbA1c, yet in 100 mg doses significantly reduces SBP in patients with T2DM. Further future research in the coming years may provide more data and information on the protective role of canagliflozin in patients with T2DM.

## Introduction

The serious complications of cardiovascular disease (CVD) in patients with T2DM exerted influence on about 451 million adults in 2017 and may increase to almost 700 million patients during 2045, thereby causing about five million deaths each year [1–3]. Based on the International Diabetes Federation (IDF) data [3, 4] the prevalence rate may be projected to rise to about 853 million by 2050. This possibly indicates 1 in 9 adults worldwide may be affected by T2DM. According to Imran et al. [5], currently, 7% or about 20.8 million adults - children of the American population is affected by T2DM. The prevalence rate of T2DM in the Iranian population has been estimated to range from10.8 -15.1% [6, 7]. To inhibit, reduce and treat the mortality outcomes in patients with T2DM death rates, canagliflozin or sodium glucose-co-transporter 2 inhibitor (SGLT2i) has been suggested to be one of the most small-molecule drug medication [1, 2, 3, 8–15 and references therein]. The drug was developed by Mitsubishi Tanabe Pharma Corporation and was approved by World Health Organization List of Essential Medicines as the trade name of Invokana (WHOLEM) in 2013 [3]. In this context, to lower the blood pressure as well as glucose level, canagliflozin was introduced as the first sodium-glucose co-transporter 2 inhibitor (SGLT2i) for the patients with T2DM, thereby decreasing the glucose reabsorption in the kidneys, increasing glucose excretion through urine and giving significant beneficial effects on the heart by reducing the risk factors of CVD, the kidney, weight management and lowering the health cost cares [8, 11–13]. Skelley et al. [15] suggested that CVD risk factors among the T2DM people are increasing, being in the range of 55–87%, but mainly relies on gender and BMI. Moreover, another anti-hyperglycemic agent (AHAs) and anti-diabetic of the SGLT2i can reduce the HbA1c by 0.5–1% [15, 16]. One of the most significant applications of canagliflozin among the patients with T2DM is related to the reductions in systolic and diastolic blood pressure, weight loss, as well as beneficial reno-protective effects [15].

Global data for T2DM patients receiving canagliflozin drug are compared to placebo-controlled randomized controlled trials (RCTs) [16]. Accordingly, the mean difference between glycated hemoglobin level, the canagliflozin and placebo groups is estimated to be about − 0.58 at 95% confidence level. The mean contrast in in body weight is evaluated to be within − 1.60 kg at 95% confidence level and the mean variation in systolic blood pressure is reported to be about − 3.93 mm Hg at 95% confidence level [15–19]. Despite few valuable systematic reviews and meta-analysis in the current literatures [8–10, 18–23], the continuing global health emergency caused by T2DM is increasing the risk and the rate of CVD morbidity and mortality in adults, thus further up to date systematic reviews and meta-analysis are merited to farther highlight the role of canagliflozin in the treatment of T2DM patients. According to recent literature survey, there are few up to dated meta-analysis reviews on the effects of cangaliflozin on CVD risk factors in T2DM patients, thus the approach in this review is to highlight the most recent advances up to 2025 in understanding the efficacy, safety, tolerability, limitation and cost-effectiveness of canagliflozin treatment compared with placebo RTCs data on CVD risk factors affecting in patients with T2DM.

## Methods

### Data sources and survey

This fixed -effects systematic review and meta-analysis were performed accordance with the Cochrane Collaboration’s recommended guidance for systematic reviews, and the research question was determined based on the Patient Intervention Comparison and Outcome (PICO format, even though we organized the review protocol according to the PROSPERO. The intervention was the prescription of canagliflozin and the comparison referred to that between T2DM patients treated with canagliflozin and a control group treated by placebo. The expected outcome was improvement in the subject’s clinical cardiovascular outcomes, including HbA1c, blood pressure and BMI. For enhancing the sensitivity of the search, we did not render any restrictions in terms of language or date of publication. General keywords were selected for each part of the PICO, and synonyms were also considered in the search. This review is conducted on the survey of the most available data appeared in Web of Science, Embase, Scopus, PubMed, Science Direct, Google Scholar, by highlighting keywords of canagliflozin, Sodium-Glucose Transport Proteins, Sodium-glucose transporter1 inhibitor (SGL1i), Sodium-glucose transporter2 inhibitor (SGL2i), Cardiovascular diseases, Diabetes Mellitus, Type 2, Obesity, Body Mass Index within 2012–2025. To search the keywords, we used “OR” between synonyms and “AND” between the main specific keywords. To increase the sensitivity of the search, any restrictions in terms of language or date of publication were not included. The general keywords were considered and included for every part of the research question as well as the synonyms. This was followed by combinations of the keywords related to independent variables that was designated as “OR” order after searching. Similar search was carried out for the keywords-related to the dependent variables. Consequently, the results were combined with one another as the “AND” order. We also searched along the gray literature such as proceedings and theses conducted on canagliflozin, but since the data were not directly related to our parameters, were not considered in the meta-analysis. The survey resulted in about 800 abstracts, methodologies, results and conclusions of the papers that were reviewed and collected. Among these, most of the irrelevant papers were excluded and the most relevant ones of about 132 papers were selected. The full texts of all studies were assessed by two authors independently, then the data were extracted with co-operation of the two authors and assessing the risk of bias was done by the two reviewers. In case of any disagreements between the authors, agreement was achieved by consulting with our statistical advisor. A method based on a numerical scale was used to evaluate the quality of articles. A checklist sourced from the Public Health Resource Unit [24] was used to this end. This checklist contains 10 questions used to evaluate placebo-controlled randomized controlled trials (RCTs) on the basis of three broad issues:

- Is the trial valid?
- What are the results?
- Will the results help locally?

**Fig. 1:**
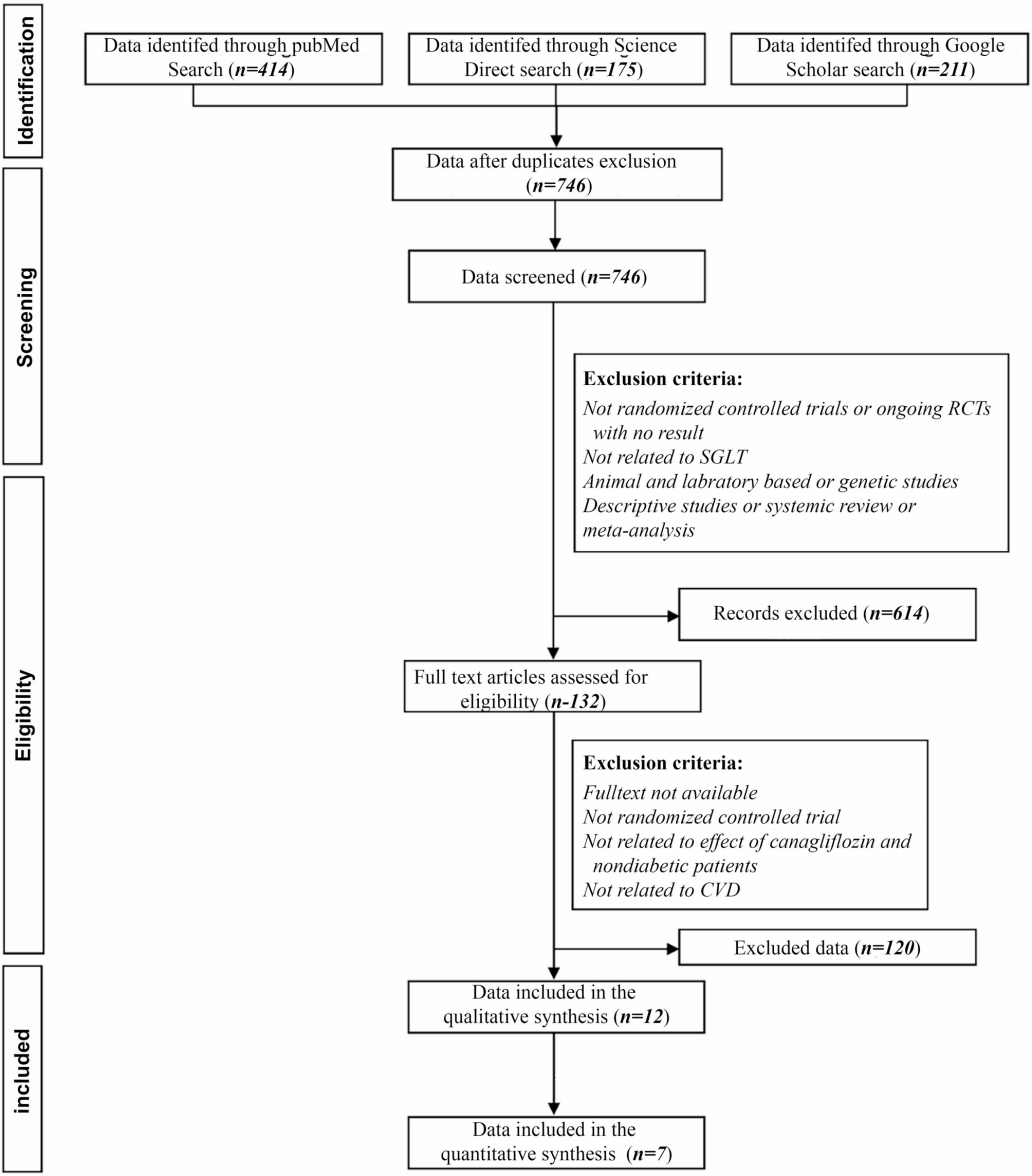
PRISMA 2009 Flow Diagram [26]

All articles were rated according to this checklist to determine those of desirable quality. Articles that achieved ratings of ≥ 6 were included in the present study. Based on our objective, we only qualified data which were already compared the effects of canagliflozin with placebo. Yet, some data were excluded as the effects of canagliflozin were compared to other anti-hyperglycemic agents. Moreover, we only focused on 100 and 300 mg prescription doses of canagliflozin, thus the data with other doses were excluded. Other exclusions were done due to the differences in displaying the methodologies and results. This process was mainly arranged by co-operation of the two authors and rarely the authors consulted with the statistical advisor. As show in flow chart (Fig. 1) only 12 qualified papers were considered for statistics data analysis of which only seven articles were included as the data synthesis (Table 1). The remaining papers were cited and discussed in the introduction and discussion sections. Statistical data analysis and interpretations were performed by Stata/SE 17.0 for Windows (Stata Corp LLC, College Station TX, USA) and Excel 2019 (Microsoft Corp., Redmond, WA, USA). The first author’s name, date of publication, quality assessment rating of each study, sample size, study group assignments, treatment duration, dose of canagliflozin, type of treatment (canagliflozin/placebo), clinical outcomes (differences in HbA1c, blood pressure and Body Mass Index) were systematically recorded.

**Table 1:** Summary of placebo and Canagliflozin clinical endpoint data

References of data	Year	Country	Sample size of Placebo	Sample size of Canagliflozin	Daily dose (mg)	Male/Female (%)	Age
Rosenstock et al. [17]	2012	USA	65	64	100	56/44	51.7 ± 0.8
			64	64	300	44/56	52.3 ± 6.9
Stenlof et al. [27]	2012	Sweden	192	195	100	41.5/58.5	55.1 ± 10.8
			192	197	300	45.2/54.8	55.3 ± 10.2
Lavalle-González et al. [28]	2013	Mexico	183	1284	100	47.3/52.7	55.5 ± 9.4
			183	1284	300	45/55	55.3 ± 9.2
Schernthaner et al. [29]	2013	Austria	755	377	300	54.9/45.1	56.6 ± 9.6
Wilding et al. [30]	2013	Unknown countries (11)	150	156	100	48.4/51.6	57.4 ± 10.5
			150	154	300	55.8/44.2	56.1 ± 8.9
Neal et al. [31]	2017	Australia	4347	5795	100	35.1/64.9	63.2 ± 8.3
Tobe et al. [32]	2024	Canada	653	875	100	31.7/68.3	59.4 ± 9.4

Estimation of each canagliflozin effect compared with placebo was expressed as standardized mean difference. The results of total variations between findings of studies (the estimations of canagliflozin effects from final studies) were carried out by means of Cochrane’s test for heterogeneity and I^2^ index. I^2^ index shows the percentage of heterogeneity between the indexes of the study. The classification of this index introduced in the Cochrane guidelines as follows, Cochrane Handbook [25]:

0‒40%: might not be important, 30‒60%: moderate heterogeneity, 50‒90%: substantial heterogeneity, 75‒100%: considerable heterogeneity. Regarding the heterogeneity of the data, all outcomes except BMI at 100 mg dose, the non-significant Cochrane’s Q test and low threshold [I^2^%] values reflect an acceptable homogeneity [26], although for BMI at 100 mg dose, only six studies indicated moderate heterogeneity. Consequently, based on our limited data, sensitivity analysis was conducted using the leave-one-out method [24]. Accordingly, the overall results were not significantly sensitive to the exclusion of any individual study, therefore the findings remained relatively stable.

## Results

Table 1 compares the effects of canagliflozin and placebo clinical endpoint data on CVD and T2DM patients reported by different authors. Accordingly, the numbers of patients in both groups are almost similar, even though the numbers of patients reported by Lavalle-Gonzales et al. [28] for the canagliflozin group are more than the placebo group by a factor of 7 and the receiving daily uptake of patients for both canagliflozin and placebo was ranged from 100 to 300 mg.

### The effects of canagliflozin and placebo drugs on the level of HbA1c

**Fig. 2:**
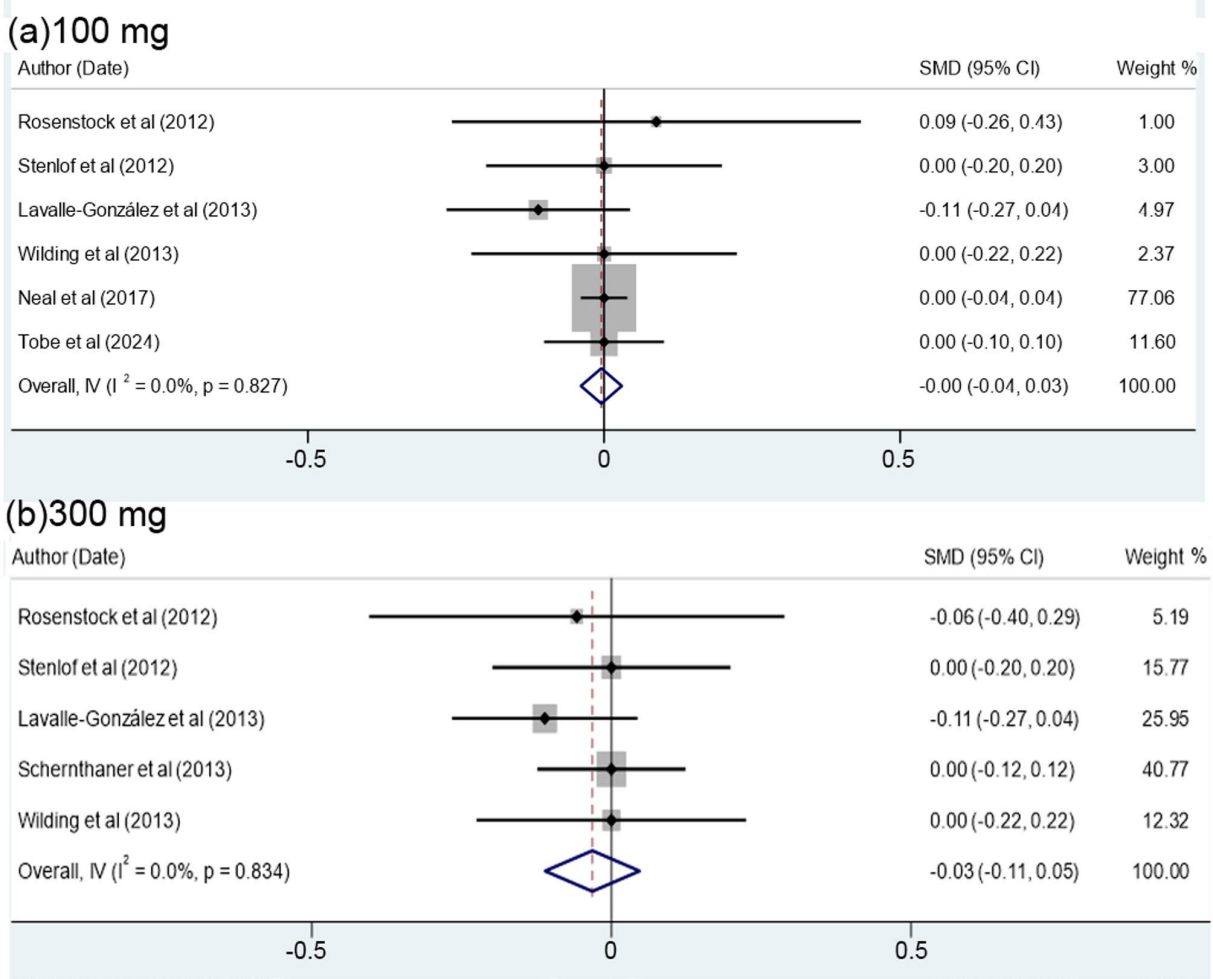
Forest plot showing the effect of canagliflozin compared to placebo on the level of HbA1c in patients receiving 100 (**a**) and 300 mg doses (**b**)

It is highly important to explore the effects of both canagliflozin and placebo on the level of HbA1c in patients with T2DM. In this regard, Table 2 shows that the effect of canagliflozin compared to placebo has led to a minor decrease in HBA1c by only- 0.01at 95% Confidence Interval of (-0.04, 0.023), even though there is no significant difference between the effect of canagliflozin data and those of placebo (*P* = 0.58). Moreover, although the daily uptake variations of the patients were in the range of 100 to 300 mg (Table 2), non-significant difference in terms of Cochrane’s Q test (*P* = 0.95) and the index of heterogeneity [(I² (% = 0.0%)] was observed. The overall results indicate that the data are homogeneous, thus projecting a heterogeneity percentage close to zero. To assess the effects of daily uptake of canagliflozin and placebo on the HbA1c level, some authors (Table 2), compared six cases of 100 mg uptake of canagliflozin to the remaining five 5 cases of 300 mg uptake of placebo. Meta-analysis of the data in Table 2; Fig. 1 demonstrates that the HbA1c level in the patients receiving 100 mg uptake was − 0.005 (Confidence Interval of 95%= -0.04 to 0.03), but show no significant difference with placebo (*P* = 0.79). In patients receiving 300 mg uptake, the cumulative effect was − 0.03 (Confidence Interval level of 95%= -0.11 to 0.05), thus did not indicate a significant difference with placebo (*P* = 0.43). As displayed in Fig. 2a, b the results obtained by meta-analysis were significantly homogeneous for both 100 mg dose (Cochrane’s Q *P* value = 0.827, I² = 0.0%) as well as for 300 mg dose (Cochrane’s Q *P* value = 0.834, I² = 0.0%).

**Table 2:** Meta-analysis data on the effects of Canagliflozin compared to placebo on the HbA1c level of blood

References of data	Year	Dose	SMD*	[95% Confidence Interval]		Weighted average (%)	Male/Female (%)	Age
Rosenstock et al. [17]	2012	100	0.09	-0.26	0.43	0.84	56/44	51.7 ± 0.8
		300	-0.06	-0.40	0.29	0.83	44/56	52.3 ± 6.9
Stenlof et al. [27]	2012	100	0.00	-0.20	0.20	2.52	41.5/58.5	55.1 ± 10.8
		300	0.00	-0.20	0.20	2.53	45.2/54.8	55.3 ± 10.2
Lavalle-González et al. [28]	2013	100	-0.11	-0.27	0.04	4.17	47.3/52.7	55.5 ± 9.4
		300	-0.11	-0.27	0.04	4.17	45/55	55.3 ± 9.2
Schernthaner et al. [29]	2013	300	0.00	-0.12	0.12	6.55	54.9/45.1	56.6 ± 9.6
Wilding et al. [30]	2013	100	0.00	-0.22	0.22	1.99	48.4/51.6	57.4 ± 10.5
		300	0.00	-0.22	0.22	1.98	55.8/44.2	56.1 ± 8.9
Neal et al. [31]	2017	100	0.00	-0.04	0.04	64.68	35.1/64.9	63.2 ± 8.3
Tobe et al. [32]	2024	100	0.00	-0.10	0.10	9.74	31.7/68.3	59.4 ± 9.4
Overall, IV (I² (%) = 0.0%, *P* = 0.95)			-0.01	-0.04	0.02	100.00		

*Standardized mean difference

### The effects of canagliflozin and placebo on body mass index (BMI)

Table 3 shows the effect of canagliflozin and placebo on the BMI. The results demonstrate that the effect of canagliflozin for both 100 and 300 mg uptake on the BMI compared to placebo was only − 0.005 (at 95% Confidence Interval of -0.04 to 0.03). In this context, there was no significant difference between the effects of canagliflozin and placebo with decreasing BMI (*P* = 0.78) (Table 3). The data in Table 3, in particular the non-significant value of Cochrane’s Q test (*P* = 0.12) suggest that the data for both canagliflozin and placebo are homogeneous. This is also supported by the index of heterogeneity (I² 34.34%), which conveys a low heterogeneity as described by Higgins [33]. This reference suggests that I² values between 0.0% and 40% are considered to be of low heterogeneity.

**Table 3:** Meta-analysis of the data on the effect of Canagliflozin compared to the placebo on the BMI

References of data	Year	Daily dose (mg)	SMD*	[95% Confidence Interval]		Weighted average (%)	Male/Female (%)	Age
Rosenstock et al. [17]	2012	100	0.24	-0.10	0.59	0.83	56/44	51.7 ± 0.8
		300	0.21	-0.14	0.56	0.83	44/56	52.3 ± 6.9
Stenlof et al. [27]	2012	100	-0.08	-0.28	0.12	2.52	41.5/58.5	55.1 ± 10.8
		300	-0.02	-0.22	0.18	2.53	45.2/54.8	55.3 ± 10.2
Lavalle-González et al. [28]	2013	100	0.20	0.05	0.36	4.16	47.3/52.7	55.5 ± 9.4
		300	0.05	-0.11	0.20	4.17	45/55	55.3 ± 9.2
Schernthaner et al. [29]	2013	300	-0.01	-0.14	0.11	6.55	54.9/45.1	56.6 ± 9.6
Wilding et al. [30]	2013	100	0.09	-0.13	0.32	1.99	48.4/51.6	57.4 ± 10.5
		300	0.08	-0.15	0.30	1.99	55.8/44.2	56.1 ± 8.9
Neal et al. [31]	2017	100	-0.02	-0.06	0.02	64.70	35.1/64.9	63.2 ± 8.3
Tobe et al. [32]	2024	100	-0.08	-0.18	0.02	9.73	31.7/68.3	59.4 ± 9.4
Overall, IV (I² =34.4%, *P* = 0.12)			-0.005	-0.04	0.03	100.00		

*Standardized mean difference

**Fig. 3:**
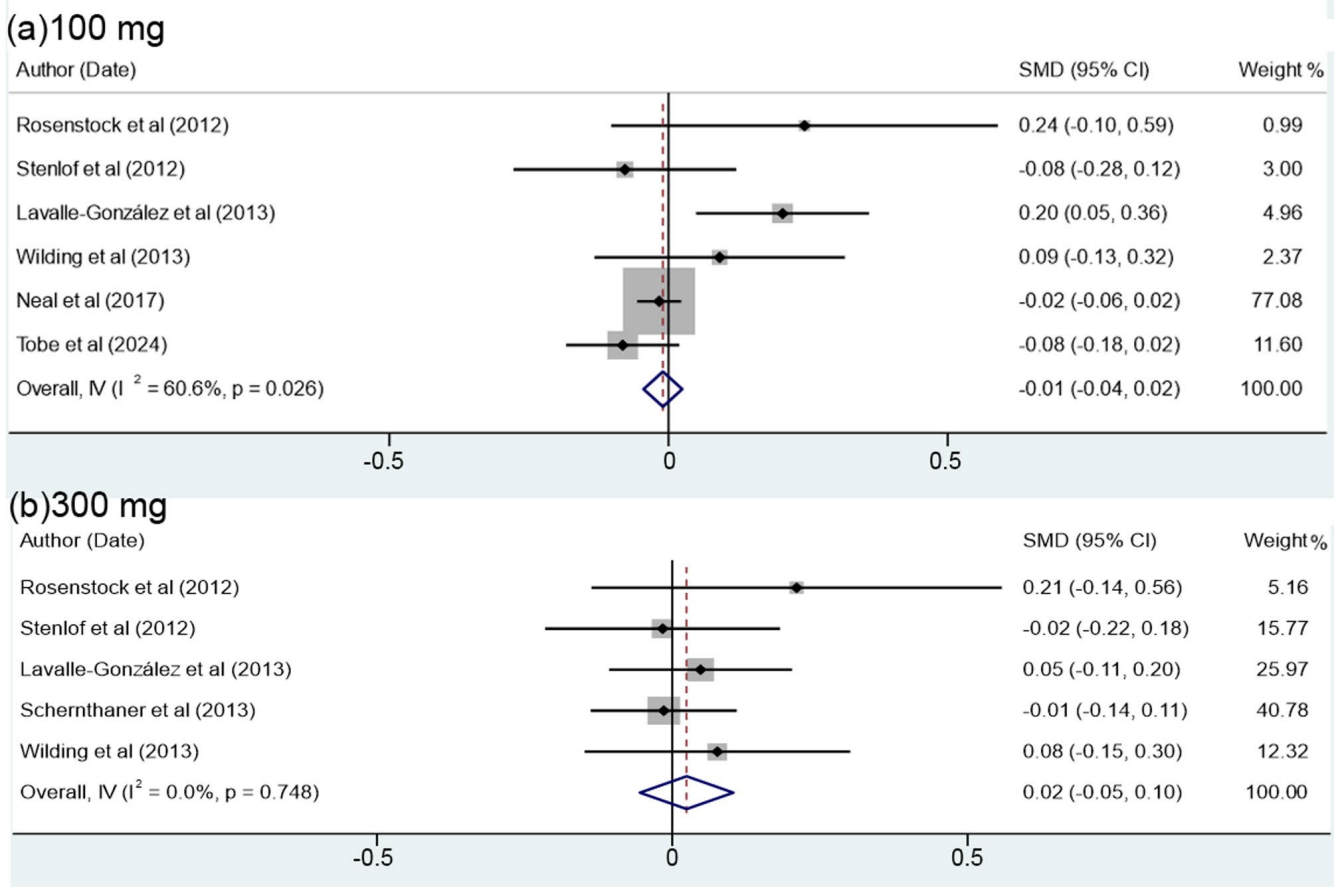
Forest plot displaying the effect of canagliflozin compared to placebo on the level of BMI in patients receiving 100 (**a**) and 300 mg doses (**b**)

The meta-analysis of the effect of canagliflozin versus BMI compared to placebo in patients receiving 100 and 300 mg uptakes is shown in Table 3; Fig. 3a, b. The results indicate that for patients receiving 100 mg uptake, the cumulative effect of the canagliflozin versus BMI was − 0.01 (at 95% Confidence Interval 95%=-0.04 to 0.02) and did not show a significant difference from that of placebo (*P* = 0.57). Importantly, for patients receiving 300 mg uptake, the cumulative effect was 0.02 (at 95% Confidence Interval=-0.05 to 0.10), being greater than that of placebo, but no significant difference was also observed (*P* = 0.55). In this particular point, for 100 mg uptake, the homogeneity of data in terms of Cochrane’s Q test (*P* = 0.02) and index of heterogeneity [(I² (%) = 60.6%] show moderate heterogeneity, thus are consistent with the findings of Higgins [25]. In contrast, for the patients with 300 doses, the *P* value for Cochran’s Q (*P* = 0.75) and the index of heterogeneity [I² (%) = 0.0%) indicate a highly homogeneous affinity.

### Effect of canagliflozin on systolic blood pressure (SBP)

**Fig. 4:**
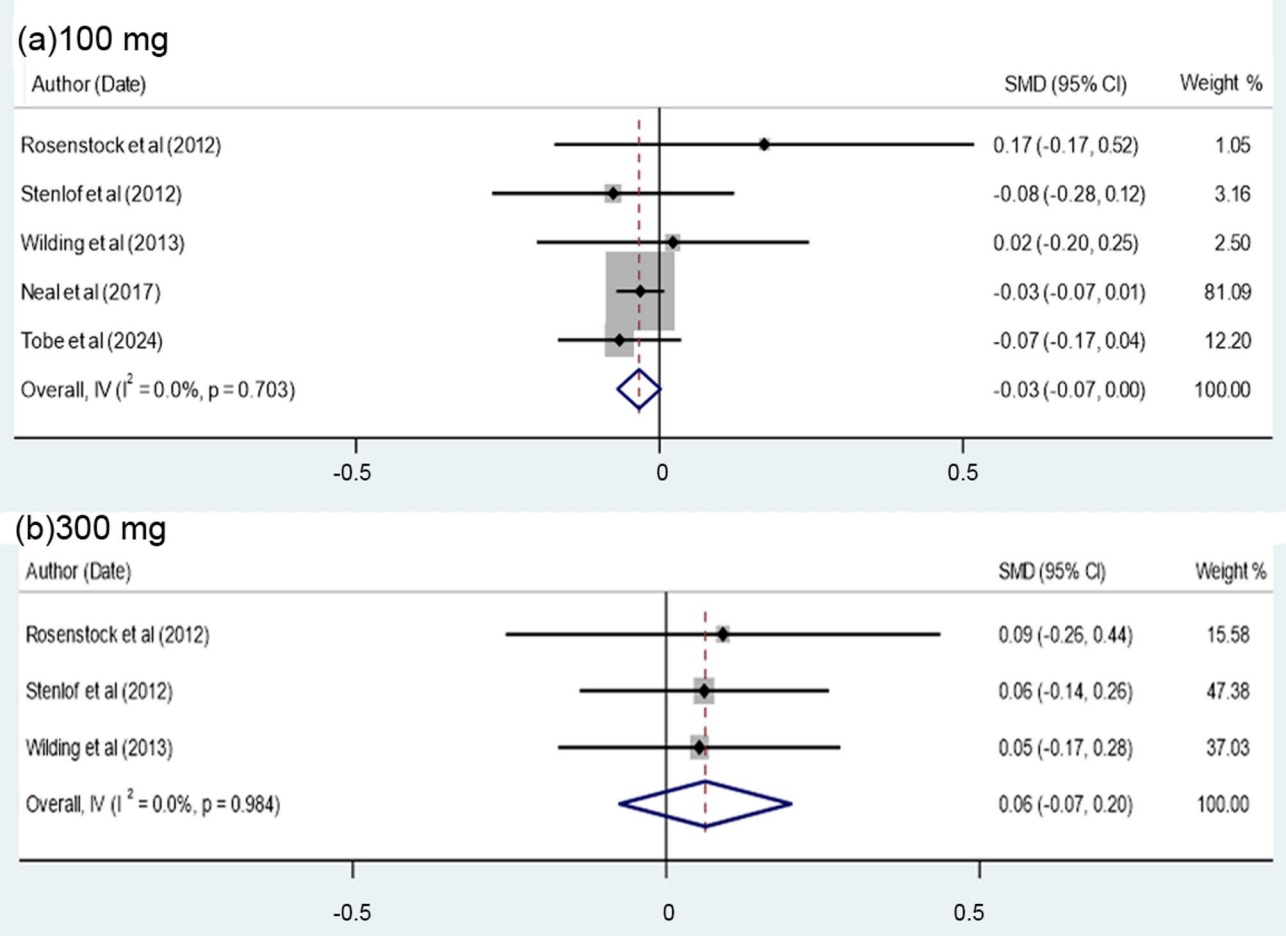
Forest plot illustrating the effect of canagliflozin compared to placebo on the level of SBP in patients receiving 100 (**a**) and 300 mg doses (**b**)

Among the 7 studies in Table 4, only 5 authors referred to the effect of canagliflozin on the systolic blood pressure of the T2DM patients. The results (Table 4) demonstrate that the cumulative effect of canagliflozin on SBP compared to placebo was only − 0.03 (at Confidence Interval of 95% = -0.06, 0.01) and show no significant difference with placebo-controlled randomized controlled trails (RCTs) data (*P* = 0.11). Based on quality control (Table 4), the data are considerably homogeneous in terms of *P* value of Cochrane’s test (Q = 0.781) and index of heterogeneity [I²=0.0%)]. More importantly, Fig. 4a, b compares the effect of canagliflozin in patients receiving 100 and 300 mg uptakes. The results of meta-analysis indicate that for the patients receiving 100 mg dose, the overall effect of the canagliflozin on SBP was − 0.03 (at 95% Confidence Interval = -0.07, 0.00) and significantly reduced SBP (*P* = 0.06). However, for the patients receiving 300 mg dose, the overall effect was 0.06 (at 95% confidence level =-0.07, 0.20) and although slightly increased the SBP level compared to the placebo, the difference is not very significant (*P* = 0.37) (Fig. 4).

**Table 4:** Meta-analysis of the data on the effect of Canagliflozin compared to the placebo on the SBP

References of data	Year	Daily dose (mg)	SMD*	[95% Confidence Interval]		Weighted average (%)	Male/Female (%)	Age
Rosenstock et al. [17]	2012	100	0.17	-0.17	0.52	0.98	56/44	51.7 ± 0.8
		300	0.09	-0.26	0.44	0.98	44/56	52.3 ± 6.9
Stenlof et al. [27]	2012	100	-0.08	-0.28	0.12	2.96	41.5/58.5	55.1 ± 10.8
		300	0.06	-0.14	0.26	2.97	45.2/54.8	55.3 ± 10.2
Wilding et al. [30]	2013	100	0.02	-0.20	0.25	2.34	47.3/52.7	55.5 ± 9.4
		300	0.05	-0.17	0.28	2.32	45/55	55.3 ± 9.2
Neal et al. [31]	2017	100	-0.03	-0.07	0.01	76.00	54.9/45.1	56.6 ± 9.6
Tobe et al. [32]	2024	100	-0.07	-0.17	0.04	11.44	48.4/51.6	57.4 ± 10.5
Overall, IV (I² =0.0%, *P* = 0.78)			-0.03	-0.06	0.01	100.00	55.8/44.2	56.1 ± 8.9
							35.1/64.9	63.2 ± 8.3
							31.7/68.3	59.4 ± 9.4

*Standardized mean difference

As indicated in Figs. 4a, b, for 100 mg dose, *P* value of Cochrane’s test (Q = 0.703) and the index of heterogeneity [ I² (%) = 0.0%)] are almost similar to that of 300 doses with *P* value of Cochrane’s test (Q = 0.98) and index of heterogeneity [I² (%) = 0.0%) with no significant heterogeneity.

## Discussion

This review discusses the effects, efficacy, safety and limitations of canagliflozin on the HbA1c, BMI and SBP variations in T2DM patients. Canagliflozin is an approved synthetic sodium-glucose co-transporter 2 inhibitor (SGL2i) therapeutic medicinal for the treatment of patients with T2DM, in particular when its efficacy has been tested by the placebo-controlled randomized controlled trials (RCTs). In this systematic review and meta-analysis, we discuss some available data to evaluate the effects of Canagliflozin at 100 and 300 mg doses on the HbA1c levels, BMI, and SBP, respectively.

Despite the effects of canagliflozin in controlling the blood sugar, this review suggests that the cumulative effect of this drug in patients with T2DM leads to a very small reduction in HbA1c of 0.01 compared to the placebo-controlled randomized controlled trials (RCTs) with the baseline of 0.14% [at 95% Confidence Interval: 0.07–0.21: 19, 39]. Moreover, the reduction in the level of HbA1c with daily dose of 100 mg and 300 mg was 0.005 and 0.03, considerably. This indicates that although the reduction in HBA1c level among patients receiving canagliflozin has a minimal effect, the data on 300 mg doses appear to have a better effect on blood sugar control [8, 9, 11, 20, 33]. Regarding the effect of canagliflozin on BMI, the data show a very small overall reduction of 0.005. Yet, the reduction in BMI with 100 mg dose reached to 0.01, but with 300 mg dose was close to that of placebo level, even though indicated a very slight increase of 0.02. Additionally, the data revealed that for the patients receiving 100 mg uptake, there was a minor significant reduction in SBP. Similar interpretations have also been suggested by previous studies [1, 2, 8–15, 18, 20].

It is reported [14, 17, 19, 33–36 and references therein] that canagliflozin is a sodium-glucose co-transporter 2 inhibitor (SGLT2i) that is considered as an authorized drug for treatment of patients with T2DM and is responsible for the majority of glucose reabsorption in the kidneys and almost all glucose is reabsorbed from the tubules [9, 11]. The maximum concentration of glucose that can be reabsorbed is referred to as the renal threshold of glucose [1, 14, 15]. Notably, canagliflozin lowers the renal threshold for glucose and significantly increases its urinary excretion, thereby reducing blood glucose concentrations in patients with hyperglycemia (HG) [8, 13, 14, 20]. Yet, some studies recognized [9, 15, 18] that SGLT1 is mainly located in the small intestine, heart, liver, lung, and kidneys at clinical dosage, whereas SGL2i is mostly detected in the kidneys. Nevertheless, Ohgaki et al. [37] suggested that interaction of canagliflozin with SGLT1 and SGLT2 led to insistently inhibited SGLT1 and SGLT2i, but with high potency and selectivity for SGLT2. More recent data [38] highlight the beneficial effects of canagliflozin in human cardiac fibroblasts (HCFs) under hyperglycemia (HG) conditions that provides protective effects on HCFs, improves mitochondrial function by restoring Ca^2+,^ thereby reducing fibroblast proliferation. It is noteworthy that the increased urinary excretion of glucose leads to mild osmotic diuresis, followed by a reduction in net calories and leads to 80–120 g weight body lose per day in patients with T2DM [14, 15, 20, 29].

With regard to the systolic blood pressure, several authors (Tables 2 and 3 in this study) suggested that the overall effect of canagliflozin on systolic blood pressure with reference to placebo-controlled randomized controlled trials (RCTs) decreased to a level of about 0.03, but was significantly reduced by 0.03 with 100 mg dose. This indicates that with 100 mg dose of the drug, more effective reduction in systolic blood pressure was yield. By receiving 300 mg dose [17, 28, 32], systolic blood pressure reached to 0.06, thus slightly increased the level of blood pressure compared to placebo, though the difference was not significant. Further studies are required to better interpret the effects of canagliflozin, particularly at the 300 mg dose on systolic blood pressure.

The data reviewed in this study demonstrate that the effect of canagliflozin and placebo on the variations of HbA1c, BMI and SBP do not present significant differences, whatsoever. Notwithstanding this, Neal et al. [31] and Tobe et al. [32] compared the effect of canagliflozin with placebo in patients with 100 mg dose of the drug. Those authors also utilized data from two trials in 10,142 participants with T2DM that had high risk of cardiovascular disease and focused on the cardiovascular and renal outcomes. In this particular point, Tobe et al. [32] evaluated the effect of canagliflozin on the variations of HbA1c, BMI, and SBP on 1,528 diabetic patients who had been diagnosed for less than 5 years. The results indicated that canagliflozin reduced the incidence of cardiovascular events and renal impairment regardless of the duration of diabetes in the patients. Neal et al. [31] pointed out that the effect of canagliflozin in patients with T2DM resulted in a lower risk of cardiovascular events compared to those receiving the placebo. Recent studies focus more on the significant effect of canagliflozin on the high-risk complications of T2DM on CVD, renal diseases and heart failure (HF) [8–10, 26, 27, 39]. Nevertheless, some studies (40) suggest that further research is merited for patients with HF and preserved ejection fraction to be certain about the possible benefit from treatment with canagliflozin.

HF in patients with T2DM is attributed to several complications and risk factors, including macrovascular and microvascular dysfunction, volume overload, kidney function impairment, and the direct effects of diabetes and insulin resistance on cardiac myocytes [9, 14, 40–43]. Nonetheless, the preservation of kidney function and the reduction of volume overload caused by SGLT2i may decrease the risk factor of HF [37]. More importantly, Rasmussen et al. [44] hypothesized that endotrophin (ETP) may be a prognostic risk marker for heart failure (HF), (CVD), kidney endpoints as well as all- cause mortality in patients with T2DM. The combined effects of canagliflozin and metformin therapy may reduce cardiovascular risk factor in T2DM patients. In this regard, Chen et al. [45] found that patients with T2DM who were treated with canagliflozin and metformin, showed a better medication safety and improvement in blood sugar, blood pressure, weight and a significant benefit in cardiovascular risk factor. Additional research [15, 46, 47] demonstrate that reno-protective effects of canagliflozin decreased hyperglycemia, whereas metformin exerted these effects without glycemic control, thereby both canagliflozin and metformin are reported to be significantly protective against the diabetic kidney disease (DKD). Thus far, the atheroprotective effects of SGLT2i on glucose, blood pressure, and obesity, are unlikely to play a significant role in the large scale [37]. Moreover, in combinational treatments, where patients with T2DM were treated with canagliflozin, the control on the level of blood sugar and weight loss may have been influenced by other medications [37]. With regard to the effects of canagliflozin on the diabetic cardiomyopathy, Yang et al. [48] reported that although canagliflozin mitigates diabetic cardiomyopathy and enhanced mitophagy in patients with T2DM, the method may show some clinical complexities in patients with T2DM. Stenlöf et al. [27] and other studies [49–51] evaluated the effect of canagliflozin as a monotherapy during 26-week period in 584 patients with T2DM and controlled their diet and exercise with placebo-controlled randomized controlled trials (RCTs).

The results demonstrated that canagliflozin significantly reduced their HbA1c and weight. In addition to monotherapy or combination therapy, other factors such as duration of patient assessment, sample size, patient’s health conditions with T2DM and the duration of diabetes may affect the effect of canagliflozin. Consequently, to determine the exact effect of canagliflozin on the mechanism of glucose reduction, long-term safety and efficacy profile of this drug as well as long-term therapeutic studies are necessary. The most limitations in this study are limited to the availability of data in age, gender, weight of the patients and inadequate data on the combinational diseases of the patients.

## Conclusion

The data presented in this review highlight that canagliflozin is an appropriate drug for treatment in patients with T2DM. It is concluded that canagliflozin at 100 mg and 300 mg doses slightly reduced HbA1c and BMI from baseline without significant difference to that of placebo. The effect of canagliflozin at 100 mg dose only marginally reduced systolic blood pressure significantly. More studies and additional data in age, gender and the effects of combinational drugs for other diseases are needed to determine the efficacy of this drug for measuring blood sugar control, weight loss, and systolic blood pressure. The main limitation of this study is related to the limited number of the available data used for meta-analysis. Other limitations may be linked to the diverse levels of the patient ethnicities as well as drugs cost for patients of the low- income populations.

## Data Availability

The original data in this review are collected and analyzed from 38 international references cited in the list of references and are not openly available except with the permission of the corresponding authors: Reyhaneh Aftabi, Email: reyhane9495@gmail.com, Hossein Aftabi, Email: haftabih@sci.uk.ac.ir.
